# Perforator Flaps for Reconstruction of Lower Limb Defects

**Published:** 2017-01

**Authors:** Mir Yasir, Adil Hafeez Wani, Haroon Rashid Zargar

**Affiliations:** Department of Plastic and Reconstructive Surgery, Skims, Srinagar, India

**Keywords:** Perforator flap, Propeller flap, Lower limb, Reconstruction

## Abstract

**BACKGROUND:**

Reconstruction of soft tissue defects in the lower third of the leg remains challenging. Anatomical constraints limit the local options available for complex defects especially lower third of leg. Local flaps based on perforator vessels are raising interest in reconstructive surgery of the limbs. We present our experience with perforator flaps for reconstruction of soft tissue defects in the lower limb.

**METHODS:**

The study was carried prospectively and 23 patients with lower limb defects treated with various perforator flaps (both elective as well as emergency) were included in the study. A hand-held ultrasound Doppler was used preoperatively and intraoperatively to detect the perforator vessels.

**RESULTS:**

Out of 23 patients, we witnessed partial flap loss in 1 and distal flap necrosis in 3 patients. Four patients had minor complications which included infection, wound dehiscence and congestion of flap.

**CONCLUSION:**

Perforator flaps may represent a good alternative to the free flaps in the areas were other local reconstructive procedures are not possible. This is a versatile technique and with decreased donor site morbidity limited to a single body area. There is a specific like to like soft tissue replacement leading to a better cosmetic and reconstructive outcome. The main drawback of the perforator flaps however is the higher risk of venous congestion.

## INTRODUCTION

Plastic surgery is a constant battle between blood supply and beauty. The end result of a reconstructive procedure is primarily attributable to the stability of the vascular component, which is fundamental in that it ensures survival and proper functioning of tissues that have been transferred to the recipient site.^1^ The lower limb has always been known for poor wound healing and, since the first steps of the plastic surgery, as a scarce source of flaps for reconstruction. Soft tissue reconstruction of the lower limb is hence, challenging. Due to limited mobility and a paucity of overlying skin, even small soft tissue defects of the lower limb generally need flap coverage.^2^

Before the introduction of microsurgery, surgeons had few reconstructive options such as local flaps (random skin flaps, muscular or musculocutancous flaps) and performed cross-legs, immobilizing the limbs for weeks.^3^ A random pattern flap has an indistinct perfusion pattern and is limited in size and mobility.^4^ Musculocutaneous flaps and muscle flaps with skin grafts such as from the gastrocnemius, soleus, and tibialis anterior can be used in the proximal and middle thirds of a pretibial defect.^5^ Unfortunately, the area least well served by these muscle flaps is the lower third of the leg. The fasciocutaneous flap reported by Ponten showed that long narrow flaps could be safely raised below the knee as long as the deep fascia was included.^6^

Ponten’s flaps were not based on specific perforators and therefore could not be islanded. Free microvascular transfer is an answer to most of the difficult reconstructions but it is time consuming, requires microsurgical facility and expertise. After a long evolution of the reconstructive methods, the reappraisal of the works of Manchot and Salmon by Taylor and Palmer opened the era of perforator flaps. This era began in 1989, when Koshima and Soeda, and separately Kroll and Rosenfield described the first applications of such flaps. Improvement in the anatomical knowledge on cutaneous, subcutaneous, and intramuscular vessels originating from major vascular axis of the limbs has allowed development of several types of perforator flaps, which today are commonly employed in clinical practice.^7^^-^^9^

With the development of perforator flaps newer and more reliable flaps have become available for lower limb reconstruction.^10^ According to the Gent consensus, perforator flaps are composed of skin and subcutaneous fat nourished by perforators arising from deep vascular systems, which reach the surface by passing mostly through muscle and intramuscular septa.^11^ Although perforator flaps technique requires microsurgical dissection, it does not require vascular suturing and can thus be defined a microsurgical nonmicrovascular flap as reported by Georgescu *et al.*^12^ Avoiding vascular sutures makes the surgical act quicker in comparison with microvascular flaps.

## MATERIALS AND METHODS

This study was conducted prospectively from August 2013 to December 2014 in the Department of Plastic and Reconstructive Surgery of Sher-I-Kashmir Institute of Medical Sciences, Srinagar, J&K, India. Twenty three patients with lower limb defects treated with various perforator flaps (both elective as well as emergency) during this period were included in the study. A written informed consent was sought from each patient included in the study.

The aetiology, site, size and characteristics of the defect and surrounding area were analysed. In designing the flaps, the vascular axes and the distribution of the perforators which could sustain them were taken into consideration. A hand-held Ultrasound Doppler was used preoperatively and intraoperatively to detect perforator vessels in the donor site area. Perforator artery selection before flap harvesting was based on vessel size and distance to the area of the defect. Once the perforator was identified, the flap was designed around the perforator or perforators according to the location and size of the defect. The dimensions of the flap were based upon the size of defect and the movement of the flap, taking into account the need to avoid excessive tension on the margins of the flap during suturing. 

The operations were performed using magnification loupes (3.5–4.0x) and microsurgical instruments. A tourniquet was inflated without prior exsanguination. This maneuver facilitates identification of perforators as they remain filled with the blood. An exploratory incision along the margin of flap was made keeping the position of marked perforator in mind. The incision is made through the skin, subcutaneous tissue, deep fascia (sub-fascial approach) and the perforator vessel is directly visualized. The incision is initially always made from one side of the flap only to properly identify and assess the calibre of the perforator. If the perforator previously identified by Doppler is not adequate, we looked for another suitable perforator and the flap design was modified accordingly. 

Careful and meticulous dissection was done in a blunt way isolating the perforator. Usually the perforator had to be dissected for several centimeters to allow easier rotation or advancement. Pedicle traction during flap harvesting and positioning was avoided. Adequate release of all fascial strands around the perforator and dissection around the perforator in intermuscular or intramuscular plane to gain additional length were then carried out. This facilitates rotation of flap without kinking the perforator. After deflation of the tourniquet, hemostasis was performed and viability of flap was evaluated. Perfusion was checked before flap rotation by waiting a few minutes and irrigating with lukewarm saline solution in order to promote microcirculation recovery. 

The flap was then rotated on its perforator to varying degrees and inset into the defect after ensuring the viability and rechecking the vascularity while in desired position. Carefully positioned drains were then applied at the end of the procedure in some patients according to need. Drains were usually removed after 24 hrs. Bandaging was soft, to avoid compression over the flap, and the limb was held in an elevated position. A window was left uncovered for monitoring of colour and temperature without bandage removal. The donor sites/secondary defects in almost all the patients were grafted. Post operative the flaps were monitored. The parameters monitored included colour, temperature, margins, signs of poor perfusion/congestion, epidermal shrinking, blistering.

## RESULTS

**Fig. 1:**
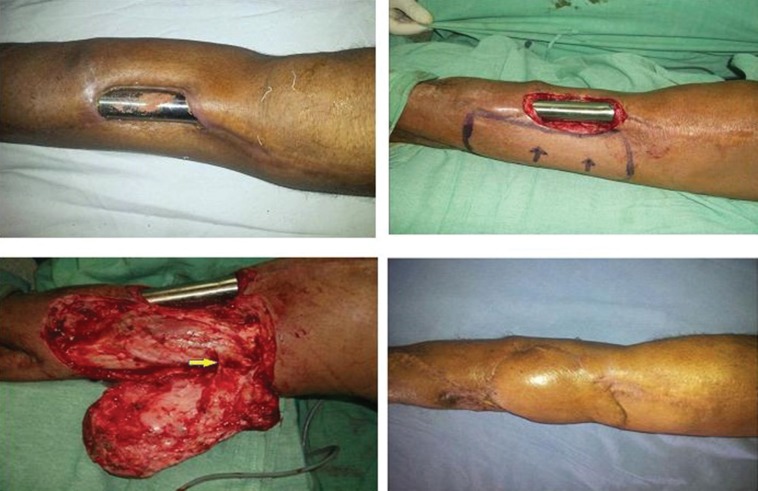
Operated case of Osteosarcoma of tibia with exposed implant. Posterior tibial artery based perforator flap used for the cover of implant

**Fig. 2:**
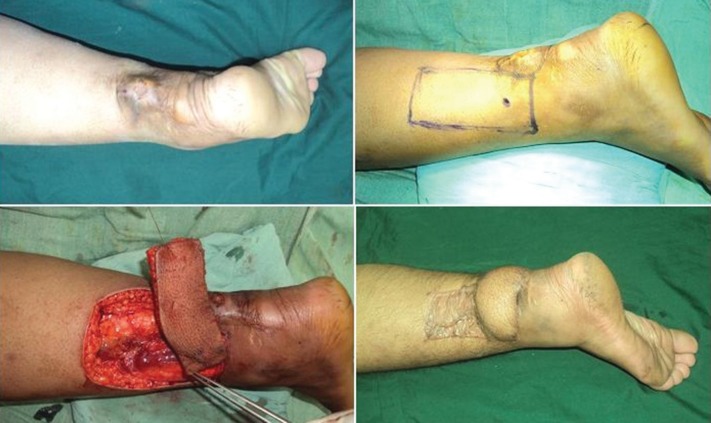
Peroneal artery based perforator flap used for the cover of post excision unstable scar defect over Achilles tendon

**Fig. 3:**
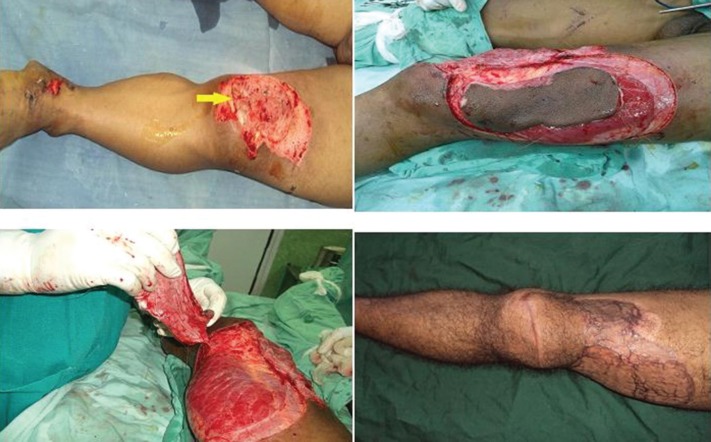
Superior genicular artery based perforator flap used for covering post RTA exposed knee joint

**Fig. 4:**
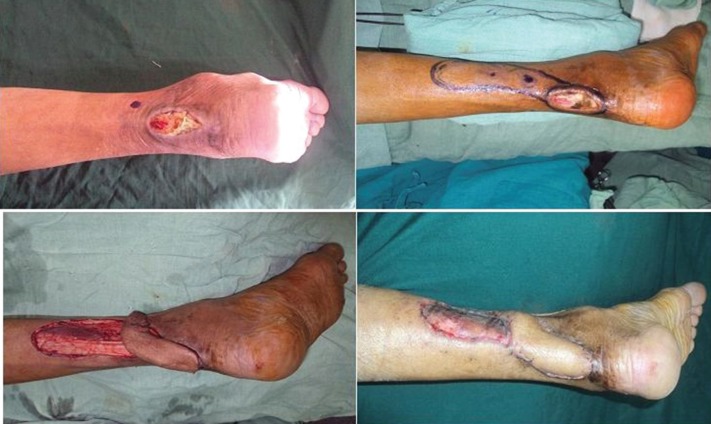
Commode injury with exposed Achilles tendon. Posterior tibial artery based ferforator flap used for cover

**Fig. 5:**
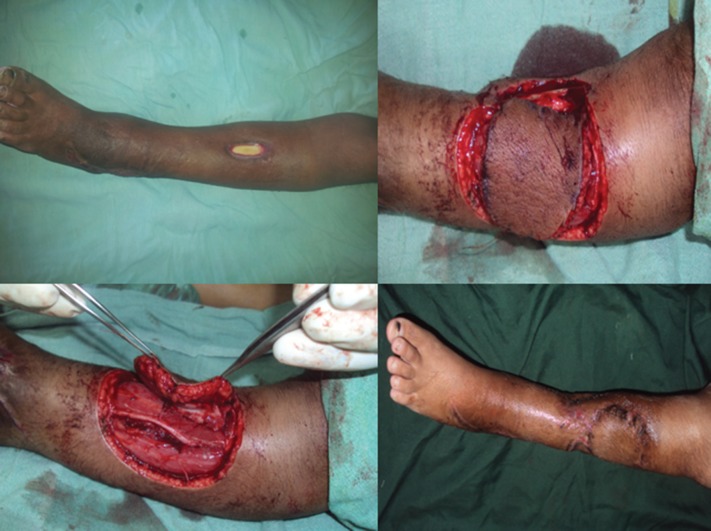
Child with cellulitis of limb with exposed Tibia following debridement, Anterior tibial artery based perforator flap cover given.

Detailed description of outcome results and complications is reported in Table 1. Almost all the various forms of perforator flaps were used in the present series. Figure 1-5 shows few index cases. In almost 50% of the cases propeller flap was used while as the other forms like perforator plus with transposition and rotation modes of movement were also used. 8 patients in our series developed complications, out of which 4 patients had minor complications which included infection, wound dehiscence and congestion of flap. These patients did not require any secondary procedure and were managed conservatively. We witnessed partial flap loss in one and distal flap necrosis in three patients. In one of these four cases, the flap was repositioned to its native site, due to suspected color changes intraoperatively and later after 3 days was successfully positioned on the desired site. The other three cases were managed with VAC and later STSG.

**Table 1:** Clinical details of patients

**S. No.**	**Age**	**Sex**	**Etiology**	**Site of defect**	**Perforator flap source artery**	**Type/ Movement**	**Number of perforators**	**Size of flap**	**Duration of procedure (hrs)**	**Donor site Management**	**Complications**
1.	24	M	Fire arm injury	R. leg (middle third)	Posterior tibial	Transposition	2	10x7	2.3	STSG	nil
2.	35	F	Unstable scar (post glass cut)	L. leg (lower third)	Peroneal	Propeller	1	8x5	2.3	STSG	nil
3.	46	M	Exposed implant (post tumour resection)	R. leg (upper third)	Posterior tibial	Rotation	2	9X6	2.3	STSG	Nil
4.	61	M	Fall from tree	R. leg (lower third)	Posterior tibial	Propeller	1	9X3	3	STSG	Nil
5	44	M	Comode injury	L. leg tend (lower third)	Posterior tibial	Propeller	1	10x6	3.15	STSG	Nil
6.	38	F	Operated S.C.C	L. gluteal region	Superior gluteal	Propeller	1	12x10	3	Primarily closed	congestion/necrosis of distal 2cm
7.	49	M	RTA	R. medial malleolus	Posterior tibial	Propeller	1	8x6	1.45	STSG	congestion/one third necrosis
8.	65	F	RTA	R. leg (upper third)	Anterior tibial	Advancement	1	5x4	1.3	STSG	Nil
9.	70	F	RTA	L. leg (lower third)	Posterior Tibial	Rotation	1	9X6	2.3	STSG	Nil
10.	55	F	Fall from tree (exposed implant)	L. leg (lower third)	Peroneal	Propeller	2	8x4	2.3	STSG	Infection
11.	45	M	Cellulitis (Infection)	R. leg (upper third)	Posterior tibial	Transposition	1	8x5	2.15	STSG	Nil
12.	60	M	RTA	L. leg (lower third)	Peroneal	Propeller	2	10x5	2.3	STSG	infection/dehiscence
13.	19	M	RTA	L. leg (middle third)	Anterior tibial	Rotation	1	9x4	1.45	STSG	Congestion
14.	50	M	Fall	R. leg (upper third)	Posterior tibial	Propellar	1	11x5	3.15	STSG	Nil
15.	55	M	Tin cut injury	L. leg (lower third)	Posterior tibial	Propeller	1	7x4	1.45	STSG	nil
16.	19	M	Trophic ulcer EX	L. heel	Posterior tibial	Propeller	1	10x5	2.15	STSG	Nil
17.	19	M	RTA	L. knee	Superior genicular	Rotation	1	15x10	3.3	STSG	congestion/necrosis of distal 2cm
18.	60	F	RTA	L. medial malleolus	Posterior tibial	Propeller	1	8x4	1.45	STSG	Nil
19.	8	M	Cellulitis	L. leg (middle third)	Anterior tibial	Advancement	2	7x5	2.15	STSG	Nil
20.	35	F	Fall	R. leg (lower third)	Posterior tibial	Propeller	1	12x6	3.3	STSG	Nil
21.	35	F	Osteomyelitis	L. leg (middle third)	Anterior tibial	Transposition	1	7x4	2.3	STSG	Nil
22.	28	M	RTA	R. leg (lower third)	Anterior tibial	Transposition	1	10x8	2	STSG	necrosis of distal 2cm
23.	70	M	Burn	R. medial malleolus	Anterior tibial	Rotation	1	6x4	3	STSG	Nil

## DISCUSSION

The lower limb has always been known for poor wound healing and soft tissue reconstruction of the lower limb is challenging.The ideal reconstruction technique for both simple and complex defects of the lower limb should replace like to like tissue, minimize donor-site morbidity, preserve main vascular trunks, and reduce operating and hospitalization time. Perforator based flaps meet most of these requirements.

The development of perforator flaps in reconstructive microsurgery has been facilitated by improved knowledge of the arterial basis of flap perfusion. The subdermic vascular network is particularly rich and allows the harvesting of thin skin flaps. One single perforator vessel located in an eccentric position in relation to a skin paddle may support a large skin area thanks to the opening of potential vascular territories, which move to the peripheral border of the flap. The process of vascular adoption is promoted by the increase of blood pressure, which occurs in the perforator artery after closure of subcutaneous and intramuscular branches during flap harvesting. One of the main characteristics of perforator flaps is their versatility, as the flap may be selected on the perforator artery according to defect type.

The present study was carried out on 23 patients who underwent reconstruction of lower limb defects with various perforator flaps. One third of our patients had soft tissue loss following road traffic accident which has shown an increase in recent years and the usual mode was motor bike accident. The various etiologies in our series included road traffic accidents, fall from height, oncological resections, post infection/cellulitis debridement, firearm injury, comode injury, unstable scar, tin cut injury, trophic ulcer and burn. Road traffic accident (35%) was the commonest of all. Maximum cases in our series as well as those reported in literature were road traffic accidents, the reason being that there has been a tremendous increase in the number of vehicles and nowadays road traffic accidents are predicted to be the third leading contributor to the global burden of disease.

The most common site of reconstruction in our study was the lower third of leg which constituted 39% of cases. The fact that lower third of leg is a difficult site for reconstruction with limited options, perforator flaps have been recommended by several clinical studies reported on the application of perforator based local flaps in lower-limb reconstruction.^13^^-^^17^ The size of perforator flap that can be safely harvested has always been a point of argument and bone of contention for plastic surgeons all over the world. The length of the flap in our study ranged from 5 to 19 cm and the width from 3 to 10 cm. The maximum size of flap harvested in our study was 19x10 cm^2 ^for covering the knee joint in a young patient with an exposed knee joint following a road traffic accident. 

The large flap territory can be raised on a single perforator due to extensive axial communications between the perforators within the flap. Hyperperfusion in a perforator allows the capture of multiple adjacent perforasomes through direct and indirect lining vessels. Panse NS *et al.*^18^ in their study made an attempt to define the safe extent of local perforator flap for lower limb reconstruction by comparing it with the limb length of the patient and concluded that there is a six times more chance that a local perforator flap will necrose if it is more than one-third of the limb length as compared to a flap which is less than one-third of the limb length. It is pertinent to mention here that there is still no standardization or reference for safe limit of a perforator flap. 

In our study, we could raise a variety of perforator flaps based on posterior tibial artery (12), anterior tibial artery (6), peroneal artery (3), superior genicular artery (1) and superior gluteal artery (1) for reconstruction of lower limb defects. In most of the cases (18) we used flaps based on a single perforator. Flaps were raised on two perforators in 5 cases. The advantage of single perforator based flaps is that this feature is best exploited by raising and rotating the flap on a single perforator. 

Maximum flaps in our series were islanded and propelled into the defect as these flaps have the advantage of maximum gain in movement and increased arc of rotation. The operative time in most of studies ranges between 2 to 3 hours. Average duration of surgery in our series was 2.30 hrs, with maximum duration of 3.15 hrs and minimum duration of 1.30 hrs. This makes the perforator flap reconstruction a preferred option for patients with co-morbidities who might not be good candidates for longer duration surgeries. 

We witnessed partial flap loss in one and distal flap necrosis in three patients following post operative venous congestion. In one of these four cases, the flap was repositioned to its native site, due to suspected colour changes and mild congestion intraoperatively and later after 72 hrs was successfully positioned on the desired site. The other three cases were managed with VAC and later STSG. the main reason for flap loss has been attributed to venous congestion which can occur due to kinking of the vein following flap rotation because of thinner wall as compared to that of the artery.

To conclude, the perforator flaps in lower limb reconstruction are a viable option in the armamentarium of a reconstructive surgeon. This is a versatile technique and with decreased donor site morbidity limited to a single body area. These flaps do not involve sacrifice of any of the main arteries. They can cover very distal defects of the leg. There is a specific like to like soft tissue replacement leading to a better cosmetic and reconstructive outcome. The operative time taken for perforator flaps is not significantly higher than that for other fasciocutaneous flaps. 

Disadvantages of these flaps are that they have a limited role in larger defects, degloving injuries, and variable location of the perforators away from the soft tissue defect can sometimes be a hurdle. The main drawback of the perforator flaps however is the higher risk of venous congestion. Because the perforator venous wall is much thinner than the perforator arterial wall, it has a greater chance of venous congestion when rotated up to 180 degrees. The proximal part of the flap which is used for defect coverage sometimes suffers from partial skin necrosis due to venous congestion. The problem of venous congestion can often be prevented with adequate dissection, loose suturing, post operative dependent drainage and massage.

Perforator flaps may represent a good alternative to the free flaps in the areas were other local reconstructive procedures are not possible. The favorable results reported in the literature, as well as the results of our personal experience for lower limb reconstruction, are encouraging. We believe that when the characteristics of the defect are suitable for treatment, this technique should be regarded as one of the possible and viable reconstructive option.
